# Geographical Weighted Regression Analysis of Hotspots of Acute Respiratory Infection and Its Associated Factors among Children <5 Years in Ethiopia: A Spatial and Multilevel Analysis

**DOI:** 10.4314/ejhs.v35i1.2S

**Published:** 2025-12

**Authors:** Yazachew Moges Chekol, Lewegneh Wegayehu Tessema, Tigabu Kidie Tesfie, Tsion Mulat Tebeje, Negalegn Byadgie Gelaw, Girum Shibeshi Argaw

**Affiliations:** 1 Department of Health Information Technical, Mizan Aman College of Health Science, Ethiopia; 2 Department of Midwifery, Gambella Teacher Education and Health Science College, Gambella, Ethiopia; 3 Department of Epidemiology and Biostatistics, Institute of Public Health, College of Medicine and Health Sciences, University of Gondar, Ethiopia; 4 Department of Epidemiology and Biostatistics, Dilla University, Ethiopia; 5 Department of Public Health, Mizan Aman College of Health Science, Ethiopia; 6 Department of public health, College of Health Science, Jigjiga University, Ethiopia

**Keywords:** Geographically weighted regression, acute respiratory infection, multilevel analysis, EDHS, Ethiopia

## Abstract

**Background:**

The prevalence of acute respiratory infection (ARI) among children under five years of age varies across geographic regions, yet previous studies have not sufficiently addressed this variation in Ethiopia. Therefore, this study aimed to examine the geographic variation of ARI in Ethiopia using spatial analysis.

**Methods:**

A total of 10,417 children under five years were included in this study. Data analysis was conducted using STATA-17, ArcGIS 10.8, and SaTScan 9.6. Variables with a p-value <0.25 in the bi-variable analysis were included in the final model, and p-values <0.05 were considered statistically significant. Ordinary least squares (OLS) and geographically weighted regression (GWR) were employed to explore the spatial relationships between the outcome and determinant variables. The model with the lowest corrected Akaike Information Criterion (AICc) value was considered the best-fit model.

**Results:**

The prevalence of ARI among children under five in Ethiopia was 12.29% (95% CI = 11.68–12.94%). Most hotspot areas were located in Tigray, central Oromia, eastern SNNPR, and southern Amhara. In spatial analysis, significant predictors of hotspot areas included a higher proportion of rural women, children with diarrhea, Muslims, women with no education, low media exposure, and the poorest households. In the multilevel analysis, secondary maternal education, child age 48–59 months, recent diarrhea, and residence in Afar, Amhara, Benishangul, and SNNP were significantly associated with ARI.

**Conclusion:**

There is notable spatial variation in the prevalence of ARI in Ethiopia. Factors such as child age, recent diarrhea, maternal education, and region were significantly associated with this spatial variation. The government of Ethiopia should re-evaluate current ARI prevention strategies and implement geographically targeted interventions to reduce the burden of ARI.

## Introduction

Acute respiratory infection (ARI) is a major public health concern caused by a heterogeneous mix of organisms that affect human airways (1). ARIs are generally classified into two major categories based on the affected anatomical region: Upper Respiratory Tract Infections (URTIs) and Lower Respiratory Tract Infections (LRTIs). The upper respiratory tract includes the airways from the nostrils to the vocal cords in the larynx and the paranasal sinuses, whereas the lower respiratory tract comprises the trachea, bronchi, bronchioles, and alveoli. ARIs are not confined to the respiratory tract and may have systemic effects due to the potential spread of infection, microbial toxins, and impaired lung function (2).

ARIs are among the most common childhood infections and represent a major global public health challenge (3). They account for approximately 20% of all deaths in children under five years of age worldwide, with Africa contributing to two-thirds of the global ARI burden (4). Children in developing countries are 10–50 times more likely to die from ARIs than those in developed countries (5). Globally, ARIs remain the leading cause of mortality and morbidity among children under five years (6). An estimated 5.4 million deaths occurred in children under five in 2017, half of which were in sub-Saharan Africa, with ARIs responsible for the highest proportion of deaths. A study conducted among children under five in 28 sub-Saharan African countries reported an overall ARI prevalence of 25.3% (7). Pneumonia alone kills more children than any other infectious disease, affecting over 700,000 children under five annually, which equates to more than 2,000 deaths per day (8). In sub-Saharan Africa, the under-five mortality rate was double the global rate in 2021, with 74 deaths per 1,000 live births compared to 38 deaths per 1,000 live births globally (9). In low-income countries, the under-five mortality rate was 67 per 1,000 live births in 2021, compared to just five per 1,000 in developed countries (9). In Ethiopia, ARIs are the leading cause of premature mortality across all age groups, particularly pneumonia, which is the main cause of death among children (10).

Multiple factors contribute to high ARI-related mortality, including poor living conditions and other socioeconomic challenges. Pediatric ARIs remain among the leading causes of morbidity in low-income countries, particularly in sub-Saharan Africa. Research indicates that ARIs in children under five are closely associated with environmental, cultural, and socioeconomic characteristics of the population (11). Previous studies have identified factors such as maternal age, maternal education, place of delivery, antenatal care (ANC) visits, vitamin A supplementation, diarrhea history, household wealth status, and child nutritional status as predictors of ARIs in children under five (12).

The likelihood of ARI among children under five varies across countries and regions. These variations can be influenced by child- and maternal-related factors, environmental conditions, and comorbid diseases such as measles, diarrhea, and malaria (13). Previous studies conducted in Ethiopia have primarily focused on the prevalence and associated factors of ARIs, without addressing their geographic distribution. Consequently, existing data fail to represent the spatial variation of ARIs in Ethiopia. Determining the geographic distribution of ARIs using geographically weighted regression is therefore critical to assess spatial variation in the relationships between determinant factors and ARI prevalence.

Geographically weighted regression analysis provides evidence on regional ARI prevalence, identifies ARI clusters, and evaluates the impact of potential risk factors. Such insights are valuable for the Federal Ministry of Health and policymakers in planning targeted interventions. A prior study in Ethiopia using the 2016 Ethiopian Demographic and Health Survey (EDHS) data assessed the spatial distribution of ARIs; however, it did not evaluate the impact of spatially varying factors or incorporate multilevel analysis to account for community-level determinants. This study, therefore, aimed to explore spatial patterns and spatially varying factors associated with ARI among children under five in Ethiopia.

## Methods and Materials

**Study design, data source, and period**: This study used the 2016 EDHS dataset, a population-based cross-sectional survey conducted from January 18, 2016, to June 27, 2016, in Ethiopia. The survey provides nationally representative estimates at the national and regional levels, as well as for urban and rural areas. Data were accessed from the Demographic and Health Survey website (https://dhsprogram.com/). Additionally, shapefiles for each enumeration area were obtained from the same source, which were essential for spatial modeling.

**Study area**: The 2016 EDHS was the fourth nationwide survey conducted in Ethiopia, the second-most populous country in Africa, with over 120 million inhabitants. Ethiopia is divided into nine administrative regions and two self-administered cities and shares borders with Eritrea, Kenya, Somalia, South Sudan, Sudan, Djibouti, and Somalia. Modern contraceptive methods are freely available to women of reproductive age at all public health facilities.

### Source and study population

**Source population**: All children under five years of age in Ethiopia.

**Study population**: Children under five years living in selected households during the survey period (January–June 2016). The Kids Record (KR) dataset was used, including a weighted sample of 10,417 children.

**Inclusion and exclusion criteria**: Children under five with short and rapid breaths during the two weeks preceding the survey were included. Children who were not alive during the interview or enumeration areas with missing longitude and latitude coordinates were excluded.

**Sampling procedures**: Interviews were conducted with 10,641 children under five who were alive at the time of the survey. A total of 635 deceased children were excluded, resulting in 10,417 eligible children for analysis (Supplementary 1). Mothers were asked whether their child experienced ARI symptoms, such as shortness of breath or fast/difficult breathing associated with chest problems, in the two weeks preceding the survey. Sample weights were applied to restore representativeness.

**Study variables**: Outcome variable: The outcome variable was the occurrence of acute respiratory infection (ARI) among children under five years of age.

**Independent variables**: Individual-level factors included child's age, religion, maternal marital status, maternal employment, maternal education, household wealth index, electricity access, vaccination status, vitamin A supplementation, breastfeeding, refrigerator ownership, maternal age, maternal occupation, child sex, place of delivery, maternal smoking status, diarrhea in the last two weeks, and media exposure.

Community-level factors included women's education, residence, region, and community media exposure. Shapefiles of Ethiopia at the regional, zonal, and district levels were obtained from the Central Statistical Agency of Ethiopia.

### Operational definitions

**Acute respiratory infection**: Children under five years exhibiting ARI symptoms, defined as short, rapid, or difficult breathing that is chest related.

**Media exposure**: Households in which members listen to the radio, watch television, or read newspapers/magazines at least once a week were considered exposed to media (14).

**Community media exposure**: If the proportion of women exposed to media in a community was below the median (≤0.1428571), it was classified as “low media exposure,” and above the median (>0.1428571) as “high media exposure.”

**Hot spot**: Areas with high ARI prevalence.

**Cold spot**: Areas with low ARI prevalence.

**Data management and analysis**: Data extraction, coding, and analysis were performed using STATA version 17, ArcGIS version 10.8, and SaTScan version 9.6. Weighted data were used to restore the representativeness of the sample. Multilevel analyses were performed due to the hierarchical nature of the EDHS data. The intra-class correlation coefficient (ICC) was calculated to assess clustering effects, with ICC >10% considered indicative of clustering.

Variables with p-values <0.25 in the bi-variable multilevel analysis were included in the multivariable regression model. Adjusted odds ratios (AORs) with 95% confidence intervals (CIs) were calculated to identify independent predictors of ARI. Statistical significance was set at p<0.05. The model with the lowest Akaike Information Criterion (AIC) was selected as the best-fitting model.

**Spatial analysis**: Spatial autocorrelation (Global Moran's I) statistics were used to assess whether the distribution of ARI was random. Moran's value close to −1 indicates dispersion, values close to +1 indicate clustering, and values near 0 indicate randomness. A statistically significant Moran's I (p<0.05) indicated non-random spatial distribution of ARIs (15,16).

Hotspot analysis was performed to detect significant clustering. Z-scores and p-values were calculated, with high Getis-Ord Gi* values indicating hotspots and low values indicating cold spots. Empirical Bayesian kriging interpolation was used to predict ARI prevalence in unsampled areas. Spatial scan statistical analysis identified likely clusters by calculating relative risk (RR) and testing for statistical significance (17).

**Spatial regression**: To examine the spatial relationships between ARI and determinant factors, ordinary least squares (OLS) and geographically weighted regression (GWR) were performed. The proportion of ARI in each enumeration area (EA) served as the outcome variable. Among the three available bandwidth methods, the adaptive kernel bandwidth was selected by minimizing the corrected AIC (AICc).

OLS was used as a global model to estimate the overall relationships between ARI and independent variables, assuming stationarity across the study area (18). GWR, a local model, was used to account for non-stationarity, allowing regression coefficients to vary by location. The Koenker test was employed to determine the appropriateness of GWR; statistical significance indicated varying relationships across locations. The best-fitting model was determined based on the lowest AICc and highest adjusted R^2^ values. Multicollinearity was assessed using the Variance Inflation Factor (VIF) (19).

## Results

A total of 10,417 children under five years were included in the analysis after applying weight samples. The mean age of participants was 3.0 years (SD ± 1.43). The largest age groups were 0–11 months (21.8%) and 48–59 months (21.0%). Most mothers (93.9%) were married. A significant proportion of participants were from the Oromia region (43.88%) and resided in rural areas (88.84%). Nearly two-thirds of mothers (65.83%) had no formal education. Regarding religion, 41.18% of respondents were Orthodox Christian. Approximately one-fourth of mothers (23.99%) were classified within the poorest wealth index category. Most children (88.22%) had experienced diarrhea recently (Tables 1 and 2).

**Table 1:** Individual level characteristics of children under 5 years, EDHS 2016 (n=10,417)

Variables	Category	Weighted Frequency (%)
Maternal age	15-19	359 (3.4)
	20-24	1960 (18.8)
	25-29	3158 (30.3)
	30-34	2383 (22.9)
	35-39	1652 (15.9)
	40-44	680 (6.5)
	45-49	225 (2.2)
Wealth index	Poorest	2499 (24.0)
	Poorer	2386 (22.9)
	Middle	2159 (22.7)
	Richer	1860 (17.9)
	Richest	1513 (14.5)
Currently working	No	7592 (72.9)
	yes	2825 (27.1)
Sex of child	Male	5342 (51.3)
	Female	5075 (48.7)
Diarrhea recently	No	9190 (88.2)
	Yes	1227 (11.9)
Age of child	0-11	2271 (21.8)
	12-23	2004 (19.2)
	24-35	1944 (18.7)
	36-47	2007 (19.3)
	48-59	2191 (21.0)
Religion	Orthodox	3579 (34.4)
	Muslim	4289 (41.2)
	Protestant	2212 (21.2)
	Others	337 (3.2)
Household electricity	No	8802 (84.5)
	Yes	1615 (15.5)
Mother education	No education	6858 (65.8)
	Primary	2807 (26.9)
	Secondary	492 (4.7)
	Higher	260 (2.5)
Place of delivery	Home	7531 (72.3)
	Institution	2886 (27.7)
Vit A supplementation	No	8251 (79.2)
	Yes	2166 (20.8)
Ever had vaccination	No	7619 (73.1)
	Yes	2798 (26.9)
Breast feeding	No	382 (3.7)
	Yes	10035 (96.3)
Marital status	Never married	169(1.6)
	Married	9781 (93.9)
	Former married	467 (4.5)
Household refrigerator	No	10084 (96.8)
	Yes	333 (3.2)
Media	yes	8524 (81.8)
	No	1893 (18.2)

**Table 2:** Community level characteristics of under-five children, EDHS 2016

Variables	Category	Weighted Frequency (%)
Region	Tigray	686(6.6)
	Afar	105(1.0)
	Amhara	1967(18.9)
	Oromia	4571(43.9)
	Somali	476(4.6)
	Benishangul	113(1.1)
	SNNPR	2169(20.8)
	Gambella	25(0.2)
	Harari	24(0.2)
	Addis Ababa	236(2.2)
	Dire Dawa	44(0.4)
Residence	Urban	1163(11.2)
	Rural	9254(88.8)
Community media	High	6152(59.1)
	Low	4265(41.0)
Community women education	Low	7215(69.1)
	High	3202(30.7)

**Prevalence of ARI**: The prevalence of acute respiratory infection (ARI) among children under five years in Ethiopia was 12.29% (95% CI = 11.68–12.94%). The prevalence varied across regions and city administrations. The highest prevalence was observed in the Oromia region (43.9%), followed by the Southern Nations, Nationalities, and Peoples' Region (SNNPR) at 20.8%, and Amhara at 18.8%. The lowest prevalence rates were reported in Harari (0.23%), Gambella (0.24%), and Dire Dawa (0.42%).

**Figure 2:**
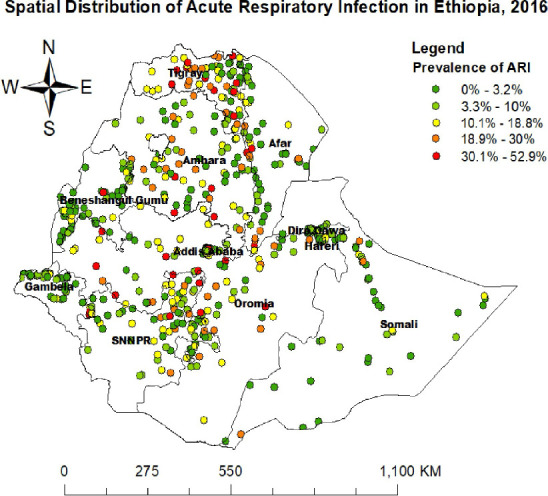
Spatial distribution of acute respiratory infection in Ethiopia, 2016

**Spatial distribution of ARI**: A total of 623 clusters were included in the spatial analysis of ARI. The map points represent clusters and their corresponding proportions of ARI. Areas with a high proportion of ARI are indicated in red, while areas with a low proportion are shown in green. The highest prevalence of ARI among children under five was concentrated in the Oromia, SNNPR, and Amhara regions (Figure 2).

**Figure 3:**
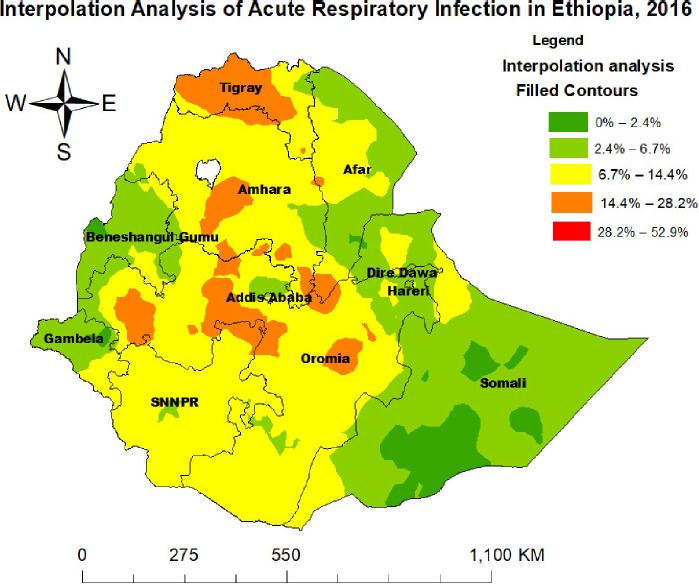
Spatial interpolation of ARI among children under 5 years in Ethiopia, in 2016

**Spatial interpolation of ARI**: Empirical Bayesian kriging interpolation was used to predict the spatial distribution of ARI. Areas shown in dark red indicate high predicted ARI prevalence, particularly in the northern, western, and southern parts of Tigray; western, eastern, and northern Oromia; and central Amhara regions. Conversely, areas depicted in green indicate the lowest predicted ARI prevalence, including the entire Somali, Benishangul-Gumuz, Gambella, Harari, and Dire Dawa regions, as well as the southern, eastern, and northern parts of the Afar region (Figure 3).

**Figure 4:**
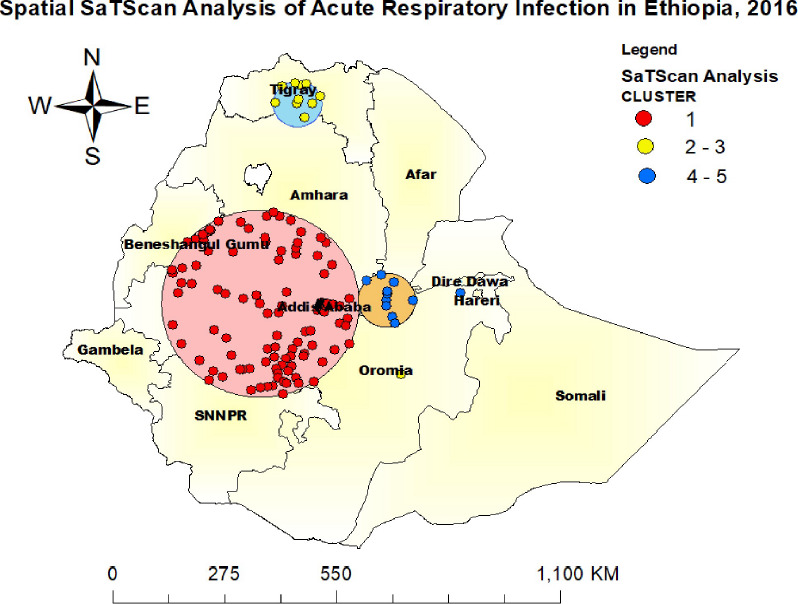
Spatial scan analysis of ARI among children under 5 years in Ethiopia, in 2016

**Spatial scan statistics of ARI**: The spatial scan statistics identified a total of 163 ARI clusters, classified as high-, medium-, and low-prevalence clusters (Figure 4). Of these, 152 clusters were primary clusters (high prevalence), accounting for 16% of all clusters; 10 were secondary clusters (medium prevalence), accounting for 26.1%; and one cluster was a tertiary cluster (low prevalence), accounting for 39.5%.

The primary clusters, indicated in red, represent the most statistically significant spatial windows and were located in the Amhara, Addis Ababa, Oromia, and SNNPR regions. The primary cluster was centered at 9.054550°N, 37.367188°E, with a radius of 239.84 km, a relative risk (RR) of 1.52, a log-likelihood ratio (LLR) of 29.37, and a p-value <0.01.

The secondary clusters, shown with yellow rings, were located in the south-central part of Tigray. This cluster was centered at 13.669272°N, 38.185383°E, with a 58.27 km radius, an RR of 2.17, an LLR of 13.42, and a p-value <0.01.

The tertiary cluster, represented by blue rings, was located in the northern part of Oromia. It was centered at 7.410925°N, 40.475708°E, with a 0 km radius, an RR of 3.25, an LLR of 10.26, and a p-value = 0.01

Ordinary Least Squares (OLS) regression analysis: The ordinary least squares (OLS) model was used to assess multicollinearity among the independent variables, and all variance inflation factors (VIFs) were below 7.5, indicating no significant multicollinearity. The OLS model explained approximately 12% of the variation in ARI prevalence (adjusted R^2^ = 0.12) with an AICc of −1,052.21.

The Koenker test was applied to assess whether the relationships between explanatory variables and the outcome were non-stationary. The Koenker statistic was statistically significant, indicating that the relationships were non-stationary or heterogeneous across the study areas. Due to the significant Koenker test, robust probabilities were used to identify significant predictors. In the OLS model, the proportion of rural residents, proportion of children with diarrhea, proportion of Muslims, proportion of women with no education, proportion of low media exposure, and proportion of the poorest women were significantly associated with ARI prevalence.

**Geographically weighted regression and nultilevel analysis**: The joint F-statistics and Wald statistics were highly significant (p < 0.01), indicating that the model was statistically robust. Spatial autocorrelation of the residuals revealed non-normal distribution (Moran's I = 0.06, p = 0.0004), suggesting that geographically weighted regression (GWR) should be applied. The Koenker statistics indicated non-stationarity, confirming spatial heterogeneity in the relationship between independent and dependent variables across the study area. The same set of independent variables used in the OLS analysis was applied in the GWR analysis.

**Geographically weighted regression analysis**: The GWR model showed improvement over the global OLS model. The corrected Akaike Information Criterion (AICc) decreased from −1,052.21 (OLS) to −1,070.23 (GWR), with a difference of −18.02, indicating that the local model better explains the spatial heterogeneity of ARI. The explanatory power of the model improved by 5%, with an adjusted R^2^ of 0.17.

In the GWR analysis, the proportion of rural women, proportion of children with diarrhea, proportion of Muslims, proportion of women without education, proportion of women with low media exposure, and proportion of the poorest women were significant predictors of ARI hotspot areas. These six factors were consistent with the significant predictors identified in the OLS model.

However, the Jarque-Bera statistics were significant, indicating potential bias in the GWR model. As a result, we did not interpret the GWR predictors further. Instead, the multilevel analysis was prioritized, as it is more robust and accounts for the hierarchical (nested) structure of the EDHS data.

**Multilevel analysis**: A mixed-effects model was conducted as a complementary approach to GWR to examine the relationships of individual- and community-level variables with ARI. Multilevel analysis accounted for the nested nature of EDHS data and analyzed the influence of individual- and community-level factors on ARI among children under five years.

The null model, which included only the outcome variable (ARI), revealed that 24.3% (95% CI = 19.9–29.2%) of the total variation in ARI prevalence was attributable to between-cluster differences. Between-cluster variability decreased across successive models: 20.5% (95% CI = 16.4–25.4%) in Model I (individual-level factors only), 16.1% (95% CI = 13.1–21.4%) in Model II (community-level factors only), and 15.6% (95% CI = 11.8–20.1%) in Model III (combined individual- and community-level factors).

Thus, the combined model incorporating both individual- and community-level factors was considered the most appropriate for predicting ARI among children under five years (Table 4).

**Table 4:** Multilevel analysis of acute respiratory infection among children under 5 years in Ethiopia, in 2016

Variables	Null model	Model I Individual level factors	Model II Community level factors	Model III Individual level and community level factors

		AOR (95% CI)	AOR (95% CI)	AOR (95% CI)
Maternal education				
No education		1		1
Primary		0.99(0.82-1.19)		0.99(0.82-1.198)
Secondary		0.59(0.39-0.88)*		0.60(0.40-0.90)*
Higher		0.74(0.43-1.285)		0.80(0.46-1.38)
Age of child				
0-11		1		1
12-23		0.95(0.76-1.19)		0.96(0.76-1.20)
24-35		0.73(0.58-0.93)*		0.74(0.58-0.94)*
36-47		0.86(0.67-1.11)		0.88(0.68-1.12)
48-59		0.59(0.46-0.78)**		0.61(0.46-0.79)**
Wealth index				
Poorest		1		1
Poorer		1.36(1.07-1.73)*		1.24(0.96-1.59)
Middle		1.47(1.13-`1.90)**		1.31(0.99-1.71)
Richer		1.39(1.05-1.85)*		1.26(0.94-1.69)
Richest		1.09(0.73-1.60)		0.95(0.62-1.47)
Religion				
Orthodox		1		1
Muslim		0.57(0.45-0.72)**		0.84(0.62-1.13)
Protestant		0.60(0.45-0.80)**		0.74(0.53-1.05)
Others		0.54(0.29-1.04)		0.66(0.34-1.28)
Region				
Tigray			1	1
Afar			0.37(0.23-0.58)**	0.46(0.27-0.79)**
Amhara			0.62(0.41-0.94)*	0.57(0.38-0.86)**
Oromia			0.83(0.57-1.21)	0.94(0.61-1.46)
Somali			0.21(0.13-0.33)**	0.28(0.16-0.48)**
Benishangul			0.11(0.06-0.21)**	0.13(0.07-0.24)*
SNNPR			0.49(0.33-0.74)**	0.54(0.34-0.87)*
Gambella			0.31(0.18-0.52)**	0.39(0.22-0.69)**
Harari			0.14(0.07-0.26)**	0.15(0.07-0.30)**
Addis Ababa			0.41(0.23-0.74)**	0.59(0.32-1.10)
Dire Dawa			0.33(0.19-0.58)**	0.38(0.20-0.71)**
Diarrhea recently				
No		1		1
Yes		4.78(3.99-5.73)**		4.78(3.99-5.70)*
Household electricity				
No		1		1
Yes		0.92(0.65-1.28)		1.06(0.74-1.51)
Household refrigerator				
No		1		1
Yes		0.70(0.44-1.12)		0.72(0.44-1.17)
Vit A supplementation				
No		1		1
Yes		1.07(0.87-1.30)		1.04(0.85-1.28)
Breast feeding				
No		1		1
Yes		1.38(0.85-2.22)		1.35(0.84-2.18)
Ever had vaccination				
No		1		1
Yes		1.19(0.97-1.45)		1.22(0.99-1.49)
Community women education				
Low			1	1
High			0.89(0.68-1.17)	0.94(0.71-1.23)
Model comparison and random effect				
ICC	0.24(0.19-0.29)	-	-	-
Deviance	5726	5336	5596	5242
AIC	5730	5380	5624	5310

**Factors associated with acute respiratory infection**: Bi-variable analysis was first conducted to identify variables significant at p < 0.25. In the bivariate regression analysis, individual-level factors such as religion, maternal education, wealth index, child age, recent diarrhea, household electricity, vitamin A supplementation, vaccination status, maternal smoking, breastfeeding, and household refrigerator ownership were significantly associated with acute respiratory infection (ARI). Community-level factors including region, residence, and community-level education were also statistically significant predictors of ARI in the bivariate analysis.

In the multilevel regression analysis, maternal education and child age were significantly associated with ARI at the individual level. At the community level, the region was significantly associated with ARI.

The overall prevalence of ARI was 8.9% (0.8–0.9%). Children of mothers with secondary education had 40% lower odds of ARI (AOR = 0.60, 95% CI = 0.40–0.90) compared to children of mothers with no education. Children aged 24–35 months had 26% lower odds of ARI (AOR = 0.74, 95% CI = 0.58–0.94), and children aged 48–59 months had 39% lower odds (AOR = 0.61, 95% CI = 0.46–0.79) compared to children aged 0–11 months. Children with recent diarrhea had 4.78 times higher odds of ARI (AOR = 4.78, 95% CI = 3.99–5.70) than those without recent diarrhea.

Regionally, children residing in Afar had 54% lower odds of ARI (AOR = 0.46, 95% CI = 0.27–0.79), those in Amhara had 43% lower odds (AOR = 0.57, 95% CI = 0.38–0.86), in Benishangul had 87% lower odds (AOR = 0.13, 95% CI = 0.07–0.24), and in SNNPR had 46% lower odds (AOR = 0.54, 95% CI = 0.34–0.87) compared to children in the Tigray region.

## Discussion

In this study, the prevalence of ARI among Ethiopian children under five years was 12.29% (95% CI = 11.68–12.94%), ranging from 0.23% in Harari to 43.9% in Oromia. This prevalence is higher than reported in rural Ethiopia (7.8%) (20), Zambia (8%) (20), and Bangladesh (4.9%) (20). Despite the introduction of combined strategies, such as the pneumococcal conjugate vaccine (PCV), ARI remains a significant public health concern in Ethiopia. The spatial distribution of ARI was non-random, with hotspot areas identified in Tigray, central Oromia, eastern SNNPR, and southern Amhara.

Conversely, the prevalence in this study was lower than in other studies conducted in Gondar Comprehensive Specialized Hospital (27.3%) (21), Northwest Ethiopia (19.2%) (22), Wolaita Sodo (21.5%) (23), Dessie (42.82%) (24), Uganda (40.3%) (25), rural India (59.1%) (26), and India (50%) (27). These differences may be due to variations in study populations, settings, age categories, outcome measurement methods, comorbidities, study period, and seasonality.

In the spatial regression analysis, significant predictors of ARI hotspots included the proportion of rural women, proportion of children with diarrhea, proportion of Muslims, proportion of women without education, proportion of women with low media exposure, and proportion of the poorest women.

Multilevel analysis revealed that individual- and community-level factors were associated with ARI. Regional differences were observed, with higher odds of ARI in Afar, Amhara, and Gambella, potentially due to cultural, environmental, and geographical factors. For instance, colder climates lead children to spend more time indoors, increasing exposure to airborne pathogens. Many households in these regions use charcoal or cow dung for fuel, which may further increase respiratory risks (27,28).

Maternal education was protective against ARI, consistent with studies in Ethiopia (28,29), Bangladesh (30), and Ghana (31). Educated mothers are more likely to recognize ARI symptoms early, seek timely healthcare, and apply knowledge about child health and hygiene (28,29,31).

Younger children (0–11 months) were more susceptible to ARI than older children (24–35 and 48-59 months), consistent with findings from other developing countries (21,32,33). This may be due to immature immunity in infants and reduced exposure to environmental pathogens.

Children with recent diarrhea were at higher risk for ARI, supported by previous studies in Ethiopia (20,35). Comorbid conditions such as diarrhea can reduce immunity, increasing susceptibility to infections like ARI (20,38,39). In resource-limited settings, poor sanitation and unsafe water contribute to the coexistence of diarrhea and ARI (40).

The use of nationally representative data, large sample size, multilevel modeling, and spatial and GWR analyses strengthens the reliability and generalizability of this study. These methods provide valuable insights for policymakers to design geographically targeted interventions.

Limitations include the use of 2016 EDHS data, which may introduce recall bias and does not allow causal inference due to the cross-sectional design. Additionally, significant predictors identified through GWR may be biased, as indicated by the Jarque-Bera test.

Despite these limitations, ARI remains a major public health issue in Ethiopia. Multilevel analysis identified child age, recent diarrhea, maternal education, and region as significant factors, while GWR analysis highlighted the proportion of rural women, children with diarrhea, Muslims, women without education, low media exposure, and poorest women as predictors of ARI hotspots. The Government of Ethiopia should evaluate current ARI prevention strategies and implement geographically targeted interventions. Further research using primary data is recommended to address the limitations of this study.

## Figures and Tables

**Figure 1:**
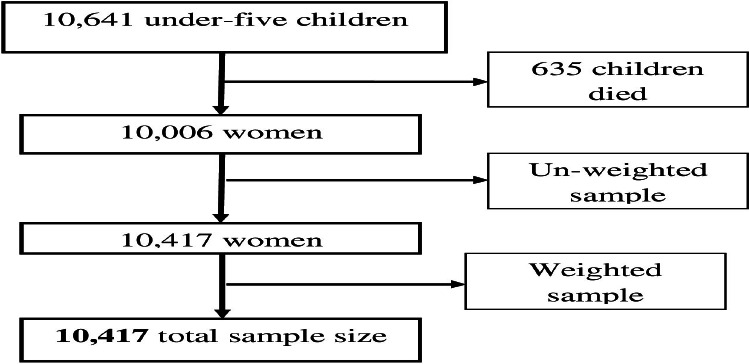
Schematic illustration of under-five children included in the study

**Table 3:** Ordinary Least Square (OLS) Regression Analysis result

Variable	Coefficient	Robust std-error	Robust t-statistics	Robust probability	VIF
Intercept	0.073	0.031	2.389	0.017170*	___
Proportion of home delivery	0.055	0.023	0.236	0.813594	4.44
Proportion of child age 24-35 month	-0.029	0.037	-0.797	0.425728	1.10
Proportion of rural women	0.049	0.015	3.341	0.000899*	3.41
Proportion of children with diarrhea	0.270	0.042	6.358	0.000000*	1.03
Proportion of children no breast feeding	0.012	0.062	0.189	0.850227	1.02
Proportion of male child	0.040	0.025	1.572	0.116524	1.03
Proportion of Muslim	-0.029	0.010	-2.966	0.003144*	1.27
Proportion of women no education	0.044	0.020	2.220	0.026747*	2.99
Proportion of women with low media exposure	-0.054	0.020	-2.573	0.010322*	3.51
Proportion of child no Vit A supplementation	-0.050	0.030	-1.692	0.091225	1.46
Proportion of poorest women	-0.033	0.016	-2.086	0.037371*	1.78
Proportion of no occupation women	0.003	0.020	0.128	0.898489	1.27
Proportion of maternal age 15-19	0.052	0.066	0.787	0.431251	1.08

**Ordinary least square regression Diagnostics.**					

Number of observations	622	Adjusted R-squared			0.12
Joint F-statistics	7.81	Prob(>F), (13,608) degrees of freedom			0.000000*
Joint Wald statistics	95.77	Prob(>chi-squared), (13) degrees of freedom			0.000000*
Koenker (BP) statistics	36.42	Prob(>chi-squared), (13) degrees of freedom			0.000510*
Jarque–Bera	228.44	Prob(>chi-squared), (2) degrees of freedom			0.000000*

VIF: Variance Inflation Factor
